# Quantification of VOC Emissions from Carbonized Refuse-Derived Fuel Using Solid-Phase Microextraction and Gas Chromatography-Mass Spectrometry

**DOI:** 10.3390/molecules23123208

**Published:** 2018-12-05

**Authors:** Andrzej Białowiec, Monika Micuda, Antoni Szumny, Jacek Łyczko, Jacek A. Koziel

**Affiliations:** 1 Faculty of Life Sciences and Technology, Wrocław University of Environmental and Life Sciences, Wrocław 50-375, Poland; micuda.monika@gmail.com; 2 Faculty of Biotechnology and Food Science, Wrocław University of Environmental and Life Sciences, Wrocław 50-375, Poland; Antoni.szumny@upwr.edu.pl (A.S.); jacek.lyczko@upwr.edu.pl (J.Ł.); 3 Department of Agricultural and Biosystems Engineering, Iowa State University, Ames IA 50011, USA; koziel@iastate.edu

**Keywords:** volatile organic compounds, torrefaction, waste to carbon, biochar, municipal solid waste, SPME

## Abstract

In this work, for the first time, the volatile organic compound (VOC) emissions from carbonized refuse-derived fuel (CRDF) were quantified on a laboratory scale. The analyzed CRDF was generated from the torrefaction of municipal waste. Headspace solid-phase microextraction (SPME) and gas chromatography-mass spectrometry (GC-MS) was used to identify 84 VOCs, including many that are toxic, e.g., derivatives of benzene or toluene. The highest emissions were measured for nonanal, octanal, and heptanal. The top 10 most emitted VOCs contributed to almost 65% of the total emissions. The VOC mixture emitted from torrefied CRDF differed from that emitted by other types of pyrolyzed biochars, produced from different types of feedstock, and under different pyrolysis conditions. SPME was a useful technology for surveying VOC emissions. Results provide an initial database of the types and relative quantities of VOCs emitted from CRDF. This data is needed for further development of CRDF technology and comprehensive assessment of environmental impact and practical storage, transport, and potential adoption of CRDF as means of energy and resource recovery from municipal waste.

## 1. Introduction

Biochar is a fine-grained product characterized by a high content of organic carbon and low susceptibility to decomposition. It is obtained in the process of torrefaction, pyrolysis, or gasification of plant biomass, biodegradable waste, and sewage sludge [1]. The European Biochar Certificate [2] defines the carbon content above 50% of dry matter as the main requirement for biochar classification. Biochar has a wide range of applications with more than 50 already documented [3]. Biochars’ intended use depends on the production process characteristics, primarily calorific value and the specific surface area [3]. The substrates used in the production of biochar include [4]: wood biomass, agricultural biomass (e.g., crop residues), energy crops (e.g., Miscanthus, energetic willow, Virginia mallow), organic waste including: organic fraction of municipal waste [5,6], waste from agro-food processing (e.g., oat fermentation, rice husks, nut shells, pomace), waste from poultry processing, animal manure, biomass from algae, digestate from biogas plants [7] and sewage sludge [8].

Municipal waste is used increasingly as a resource to recover energy and materials via thermal processes. The direction being actively pursued in the field of torrefaction and pyrolysis of municipal waste is the conversion of the fraction of combustible fraction of waste (a.k.a. refuse-derived fuel; RDF) into high-calorific solid fuel (CRDF) as a ‘Waste-To-Carbon’ waste management strategy [5,9].

One of the challenges related to the development of torrefaction and pyrolysis technology for municipal waste is the expected potential environmental impact of biochar through emissions of volatile organic compounds (VOCs). The VOCs are defined as any organic compound with an initial boiling point less than or equal to 250 °C measured at a standard pressure of 101.3 kPa [10], i.e., capable of off-gassing potentially hazardous compounds during production, storage, transportation, and use. This working hypothesis is derived by analogy to previous studies on the qualitative and quantitative analysis of VOCs content in biochars from biomass. From the research on other types of (pyrolyzed) biochar conducted so far, the occurrence of up to 140 [11] VOCs was observed, of which 74 were identified. The most frequently observed compounds in biochar from pyrolysis were acetone, benzene, methyl ethyl ketone, toluene, methyl acetate, ethanol, phenol, and cresols. Buss et al. [12] reported elevated levels of aliphatic acids and naphthalene. The ‘Char Team 2015’ reported 26 VOCs [13].

The problem of VOCs emissions from biochar was also reported by Taherymoosavi et al. [14], who analyzed biochar from compost. Particular attention has been paid to the generation of VOCs from the BTEX group in biocarbon as compared to raw materials. The content of VOCs in biochar depends on substrates as well as the process in which the char is produced [15]. Spokas et al. [11] compared the processes of biocarbon formation in terms of VOC content. For this purpose, biochar originating from various substrates, e.g., coconut, hardwood, and pig manure, produced at various process temperatures from 200 °C to 800 °C were subjected to analyses on GC-MS. A relationship was observed that the higher the temperature of the biochar formation process, the smaller the amount of VOCs emitted [11]. The highest number of absorbed VOCs is observed in the biochar derived under hydrothermal carbonization and rapid pyrolysis. These were primarily furans and aldehydes. Similar conclusions came from Wang et al. [15] analyzing the content of PAHs in biocarbon. The lowest concentration of PAH was observed in slow pyrolysis and longer retention (inside reactor) time. Thus, it is reasonable to expect that a torrefied (i.e., low temperature) process used for RDF production will result in greater VOC emissions. 

To date, published literature on VOC emissions focuses on biochars produced from biomass, mainly via pyrolysis. However, no VOC emissions have been evaluated from biochars produced from municipal waste, and in particular from torrefied RDF, a new potential future fuel source in a circular economy. For this reason, the main purpose of the work was to identify VOCs emitted from carbonized-RDF (CRDF) biochar produced via torrefaction from RDF and quantify their emissions. Information is needed about the types and quantities of VOCs emitted. This, in turn, can address many practical questions about the potential toxicity; storage, transport, and adoption of CRDF as a future energy source.

## 2. Results

In this work, for the first time, emissions of VOCs from CRDF was studied qualitatively and quantitatively. Qualitative analysis consisted of identifying compounds based on MS spectral database and available literature (Kovats Retention C7-40 Index). Table 1 shows the VOCs emitted from the analyzed CRDF with the GC column retention time and the coefficient both in the literature and with the GC software presented in the database (Kovats Retention C7-40 Index). Also included was the internal standard (2-undecanone) added during analyzes (compound #80). 

The 84 VOCs (without internal standard) have been identified. These compounds belong to various groups such as alcohols (e.g., pentanol), aldehydes, (e.g., nonanal, octanal, heptanal, hexanal, furfural), ketones (e.g., heptanone), aromatic compounds (including toluene and benzene derivatives), polycyclic aromatic hydrocarbons (PAHs; including naphthalene derivatives considered toxic), acids (e.g., acetic, benzoic), alkenes (e.g., styrene), phenols and a large group of heterocyclic compounds (including pyridine and pyrazine with derivatives). 

The largest (by number) group were derivatives of benzene and naphthalene (e.g., tetralin). The highest density of peak elution of VOCs from the chromatographic column occurred between 7 to 12 min (Table 1). Most of the identified compounds had boiling points between 100 and 240 °C; i.e., the typical range of VOCs [16]. One compound was classified as very volatile (VVOCs) and one as a semi-VOC (Table 2). Among the identified compounds, many have been known to have a negative impact on human health and the natural environment, including mutagenic and carcinogenic aromatic compounds, e.g., toluene, benzene, ethylbenzene or cumene, and PAH, e.g., naphthalene.

The total mass of VOCs emitted from CRDF was 16.4 mg/kg (Table 2) based on 7 days of accumulation in the headspace of a sealed storage vessel. The top 10 compounds with the highest emissions were as follows: nonanal, octanal, heptanal, butylbenzene, hexanal, 1-methyl-4-prop-1-en-2-ylcyclohexene, benzaldehyde, decanal, toluene, and hexylbenzene. Among the analyzed compounds, the highest emission (as a group) from the CRDF was determined for aldehydes: nonanal, followed by octanal, and heptanal (Table 2). The top 10 of the most emitted VOCs consisted almost 65% of total emissions.

## 3. Discussion

The determined composition of the VOCs mixture emitted from CRDF stored in a sealed vessel (this research) is unique because it was likely driven by the type of municipal waste and the process parameters used for its production. However, for illustrative purposes, it is useful to compare with VOCs emitted from other types of biochar. Spokas et al. [11] reported 140 different compounds, 74 were identified in all studied biochars, generated from 77 different materials; but without municipal solid waste and without fuels derived from municipal waste. Spokas et al. [11] have not found clear feedstock dependencies to the adsorbed VOC composition, suggesting a stronger linkage with biochar production conditions coupled with post-production handling and processing. Lower pyrolytic temperatures (≤350 °C) produced biochars with adsorbed VOCs consisting of short carbon chain aldehydes, furans, and ketones; elevated temperature biochars (>350 °C) typically were dominated by adsorbed aromatic compounds and longer carbon chain hydrocarbons. 

In the present work, only eight compounds were also reported by Spokas et al. [11] (Table 2). This relatively small number of common VOCs corroborates the unique influence of feedstock type —CRDF (in this research), and torrefaction process (a lower temperature process different to pyrolysis, and gasification) on VOCs formation during waste/biomass thermal treatment. Similarly, to present studies [11] aldehydes were identified in biochars (Table 2).

Buss et al. [12] analyzed VOCs emitted from three algal biochars, including two contaminated by re-condensates during pyrolysis. Buss et al. [12] identified numerous compounds from phenol groups mainly methylated and ethylene (25 compounds, but only phenol was common with present study) and acids such as acetic, formic or propionic. Taherymoosavi et al. [14] used municipal waste (compost) for the production of biochar and thus, was closest (as a source) to this work. Taherymoosavi et al., [14] analyzed biochar formed in the pyrolysis process at temperatures from 105 to 650 °C and reported the presence of alkylbenzenes, methoxy alkylphenols, organic compounds containing nitrogen, furans, and aromatic compounds. However, only phenol was a common compound identified in the present study (Table 2). Compared results show that only two compounds acetic acid and phenol were identified in the present study and [11,12], and [12,14] respectively.

There is little research in literature related to the subject of qualitative and quantitative identification of VOCs emitted from the surface of biochar, especially from biochar produced from municipal solid waste such as CRDF. This is a relatively new topic related to the trend of using torrefaction, and low-temperature pyrolysis of municipal solid waste in recent years. These new trends in municipal solids treatment are being sought as an alternative to both energy production and ‘Waste to Carbon’ utilization (e.g., CRDF). Thus the interest in identifying and mitigating VOC emissions from biochar will likely increase. As biochar VOCs are still not deeply explored, it is required to continue research on the effects of feedstock type and thermal treatment conditions on VOCs formation and emission, especially in the contest on potential harmful effect to workers during biochar storage and transportation and end users.

## 4. Materials and Methods 

### 4.1. CRDF Used in the Experiment

CRDF was produced in the torrefaction process at 260 °C and a 50 min retention time in a batch reactor, according to the procedure described by [5]. The analyzed CRDF from the torrefaction of municipal waste at 260 °C and 50 min of retention time was characterized by physicochemical properties similar to those described in the literature. CRDF with a lower heating value (LHV) of 25.95 MJ/kg was similar to CRDF obtained in earlier studies [5] and to biochar from grass produced in a similar temperature range (250 to 350 °C) by Weber and Quicker [17], which had a calorific value of 25 to 30 MJ/kg. The higher heating value (HHV) of CRDF used in this experiment (27.315 MJ/kg) could define it as a ‘hard coal’ (HHV > 23.9 MJ/kg), according to the IEA’s classification [18]. The moisture content of the analyzed material (1.54%) was in the 1 to 6% range [19]. The proximate and ultimate properties of the CRDF used were summarized by Białowiec et al. [9]. 

### 4.2. Qualitative and Quantitative Analyses of VOC Emitted from CRDF

Measurements of VOCs were made using headspace (HS) solid-phase microextraction (SPME) technology for gas extraction and gas chromatography coupled with mass spectrometry (GC-MS) (Palo Alto, CA, USA) for analyses. SPME technology combines sampling and sample preparation and is suited for exploratory qualitative and quantitative work on VOC emissions from a wide range of sources such as contaminated soils [20,21], decaying animal carcasses [22,23], fermentation by-products in beverages and aromas in wines [24,25], biological fluids and gases [26,27,28,29,30]. A comprehensive review of SPME applications to food and environmental analysis was published by Merkle et al. [31]. The apparatus and reagents were as follows:

1) the internal standard—a solution of 2-undecanone at a ratio of 20 μg compound per 20 mL of distilled water;
2) water bath with a temperature of 40 °C with glycol;
3) manual holder for SPME;
4) universal SPME fiber 3-component DVB/CAR/PDMS 50/30 μm coating (Supelco Inc., Bellefonte, PA, USA);
5) 10 μL syringe for internal standard addition;
6) a laboratory incubator (Thermo Fisher Scientific Inc., Waltham, MA, USA) with a constant temperature of 23 °C.

### 4.3. Preparation of CRDF Samples

To prepare the samples for VOCs emission analysis, the CRDF was pre-treated and ground in a 2SIEL90L2 grinding mill (Celia Indukta, Bielsko-Biała, Poland) to homogenize the sample to size <0.5 mm. Next, 10 g of bulk 3 subsamples were placed in a sealed 1000 mL glass vessels. An internal standard, 10 µg of 2-undecanone (Sigma-Aldrich, St. Louis, MO, USA), was added to the vessels to account for the variability in emissions and to aid VOC quantification. Each sealed sample was stored in a laboratory incubator at a constant temperature of 23 °C for 7 days, after which it was removed for sampling. The VOCs extraction was carried out from the headspace of sealed vessel, by the SPME.

### 4.4. Solid-Phase Microextraction 

After placing the sealed vessel with the sample in a water bath with glycol preheated to 40 °C, a 3-component universal fiber coating (DVB/CAR/PDMS 50/30 μm) was introduced into the vessel headspace. The SPME exposure lasted 20 min, similarly to the types of coatings and extraction times used for VOC emissions from solid, porous matter. The DVB/CAR/PDMS 50/30 μm SPME coating is often recommended and used for exploratory work on VOC emissions from unknown sources [25,26,28]. The coating represents a mixture of polymers capable of extracting VOCs with a wide range of properties, i.e., suitable for the work with CRDF. No specific optimization was made on sampling time. However, it was chosen based on practical considerations and preliminary trials aiming at reliably extracting the greatest number of VOCs in a relatively short extraction. 

### 4.5. Gas Chromatography with Mass Spectrometry

The separation, identification and quantification of VOCs adsorbed on the fiber was conducted using a GC coupled to a MS detector (Saturn 2000 MS Varian Chrompack, Palo Alto, CA, USA) with ZB-5 (Phenomenex, Torrance, CA, USA) column (30 m × 0.25 µm film ×  0.25 mm i.d.). Chromatographic conditions were performed according to Calin-Sanchez et al. [32]. Scanning (1 scan/s) was performed in the range of 35–400 *m*/*z* using electron impact ionization at 70 eV [33]. The analyses were performed using helium as a carrier gas at a flow rate of 1.0 mL/min, in splitless mode in SPME, and with the following program for the oven temperature: 50 °C at the beginning; 4 °C/min to 130 °C; and 10 °C/min to 180 °C and 20 °C/min to 280 °C with a hold for 4 min. The injector was held at 220 °C.

### 4.6. Data Analysis 

The VOCs emitted from CRDF samples were identified using three independent analytical methods: retention indices (RI), GC–MS retention times of authentic chemical standards, mass spectra of compounds [34] and comparison with authentic standards, if possible.

The retention index standards used in this study consisted of a mixture of aliphatic hydrocarbons ranging from C-7 through C-40 dissolved in hexane [34].

The use of internal standard enabled quantitative analysis of VOCs. It was carried out using the Mnova MS 12.0.1 software (Mestrelab Research, S.L., Santiago de Compostela, Spain) based on the retention time of individual compounds, through the integration of the peak area of the chromatogram. The percentage ratio of individual VOC was determined. VOC emissions (on per mass of CRDF basis) were estimated based on the recovered internal standard. All raw data were shown as Supplementary Materials.

## 5. Conclusions

In the analyzed CRDF (biochar) from municipal waste, 84 VOCs have been identified, including many that are toxic, e.g., derivatives of benzene or toluene. The highest emission was measured for nonanal, octanal, heptanal. The top 10 of the most emitted VOCs consisted almost 65% of total emissions. The mixture of emitted from CRDF VOCs differed from those emitted by other types of biochars, produced from different types of feedstock, and under different pyrolysis conditions. SPME provided a useful tool for characterizing VOC emissions from CRDF, a new potential fuel exemplifying the ‘Waste to Carbon’ concept in a circular, zero-waste economy.

## Figures and Tables

**Table 1:** VOCs emitted from torrefied carbonized refuse-derived fuel (CRDF).

#	Retention Time (min)	Compound Name, IUPAC	Retention Coefficient, KI Experimental (MS Database)	Retention Coefficient (Kovats C7-40 Index)	CAS Number
1	1.87	acetic acid	-	593	123-72-8
2	2.45	propanoic acid	700	700	79-49-4
3	2.93	pyrimidine	740	736	289-95-2
4	3.10	pyridine	753	746	110-86-1
5	3.29	pentan-1-ol	768	765	71-41-0
6	3.36	toluene	774	769	108-88-3
7	3.45	2-methylpropanoic acid	781	775	79-31-2
8	3.78	hexanal	804	800	66-25-1
9	4.23	2-methylpyrazine	826	831	109-08-0
10	4.41	furan-2-carbaldehyde	835	833	98-01-1
11	5.01	1,3-xylene	864	866	108-38-3
12	5.06	2-oxopropyl acetate	866	870	592-20-1
13	5.18	1,4-xylene	872	866	106-42-3
14	5.35	pentanoic acid	881	902	109-52-4
15	5.49	unknown compound	887	-	
16	5.63	heptan-2-one	893	891	110-43-0
17	5.68	styrene	896	893	100-42-5
18	5.78	1,2-xylene	900	887	95-47-6
19	5.88	heptanal	904	902	111-71-7
20	6.03	hexa-2,4-diene, (*E,E*)-	909	911	592-46-1
21	6.15	1-(furan-2-yl)ethanone	914	912	1192-62-7
22	6.24	2-ethylpyrazine	917	921	13925-00-3
23	6.34	2,5-dimethylpyrazine	920	925	123-32-0
24	6.55	cumene	927	926	98-82-8
25	6.64	1,4-dimethylpyridine	931	930	108-47-4
26	6.81	4,6,6-trimethylbicyclo[3.1.1]hept-3-ene	936	937	80-56-8
27	6.97	3-methylbutanoic acid	942	947	503-74-2
28	7.02	4-ethylpyridine	944	956	536-75-4
29	7.34	*n*-propylbenzene	955	953	103-65-1
30	7.53	benzaldehyde	962	963	100-52-7
31	7.59	5-methylfuran-2-carbaldehyde	964	965	620-02-0
32	7.77	1,3,5-trimethylbenzene	970	972	108-67-8
33	8.14	phenol	980	983	108-95-2
34	8.47	4-methyl-1-propan-2-ylcyclohexene	993	988	500-00-5
35	8.53	1,2,4-trimethylbenzene	996	993	95-63-6
36	8.79	octanal	1005	1003	124-13-0
37	8.87	dec-3-yn-1-ol	1007	1011	51721-39-2
38	9.06	an unknown isomer of ethyldimethyl benzene	1013	-	-
39	9.45	1,3-diethylbenzene	1025	1025	141-93-5
40	9.50	1-methyl-4-propan-2-ylbenzene	1027	1026	99-87-6
41	9.61	1-methyl-4-prop-1-en-2-ylcyclohexene	1030	1031	138-86-3
42	9.87	2,3-dihydro-1*H*-indene	1037	1030	496-11-7
43	10.32	1,2-diethylbenzene	1051	1045	135-01-3
44	10.42	1-methyl-2-propylbenzene	1055	1047	1074-17-5
45	10.53	butylbenzene	1058	1054	104-51-8
46	10.61	1-ethyl-3,5-dimethylbenzene	1060	1058	934-74-7
47	10.70	2-ethyl-1,4-dimethylbenzene	1063	1071	1758-88-9
48	10.87	1-phenylethanone	1068	1065	98-86-2
49	11.23	2-ethyl-1,3-dimethylbenzene	1079	1080	2870-04-4
50	11.29	4-ethyl-1,2-dimethylbenzene	1081	1083	499-75-2
51	11.36	1-ethenyl-2,4-dimethylbenzene	1083	1084	2234-20-0
52	11.54	2-ethyl-1,4-dimethylbenzene	1089	1090	1758-88-9
53	11.63	2-methoxyphenol	1091	1090	90-05-1
54	11.70	1-undecyne	1093	1095	2243-98-3
55	11.84	methyl benzoate	1098	1095	93-58-3
56	12.00	undecane	1102	1100	1120-21-4
57	12.05	nonanal	1104	1103	124-19-6
58	12.25	1,2,4,5-tetramethylbenzene	1110	1116	95-93-2
59	12.33	an unknown isomer of diethylmethylbenzene	1113	-	-
60	12.54	unknown compound	1118	-	-
61	12.68	1,2,3,5-tetramethylbenzene	1122	1117	527-53-7
62	12.94	1,3-dimethyl-2,3-dihydro-1*H*-indene	1130	1135	4175-53-5
63	13.33	5-methyl-2,3-dihydro-1*H*-indene	1142	1136	874-35-1
64	13.49	1,3-diethyl-5-methylbenzene	1145	1147	2050-24-0
65	13.70	4-methyl-2,3-dihydro-1*H*-indene	1152	1148	824-22-6
66	13.90	1-methyl-1*H*-indene	1158	1157	767-59-9
67	13.94	pentylbenzene	1160	1158	538-68-1
68	14.08	1,2,3,4-tetrahydronaphthalene	1163	1157	119-64-2
69	14.14	1,4-diethyl-2-methylbenzene	1165	1164	13632-94-5
70	14.28	2,4-diethyl-1-methylbenzene	1168	1166	1758-85-6
71	14.83	azulene	1185	1182	275-51-4
72	14.99	1-methyl-4-propan-2-yl-2-[(*E*)-prop-1-enyl]benzene	1190	1191	97664-18-1
73	15.18	2-ethyl-2,3-dihydro-1*H*-indene	1196	n.d.	56147-63-8
74	15.52	decanal	1203	1206	112-31-2
75	15.70	unknown compound	1212	-	-
76	17.42	hexylbenzene	1253	1260	1077-16-3
77	17.57	6-methyl-1,2,3,4-tetrahydronaphthalene	1266	1263	1680-51-9
78	17.66	5-methyl-1,2,3,4-tetrahydronaphthalene	1269	1276	2809-64-5
79	18.17	4,7-dimethyl-2,3-dihydro-1*H*-indene	1284	1282	6682-71-9
80	18.57	undecan-2-one (internal standard)	1296	1298	112-12-9
81	18.77	2-methyl-5-propan-2-ylphenol	1302	1299	499-75-2
82	19.11	1-methylnaphtalene	1314	1307	112-44-7
83	19.43	3,3-dimethyl-2*H*-inden-1-one	1325	1330	26465-81-6
84	19.70	1,5-dimethyl-1,2,3,4-tetrahydronaphthalene	1334	1341	21564-91-0
85	20.82	5,6-dimethyl-1,2,3,4-tetrahydronaphthalene	1373	1381	21693-54-9

**Table 2:** VOCs emissions (accumulated in a headspace of sealed vessel over 7 days of storage) from (torrefied) carbonized refuse-derived fuel ordered from the highest (µg of VOC per kg of CRDF) to lowest; % of total emissions, boiling point, VOC classification, and a comparison with VOCs emitted from other types of (pyrolyzed) biochar (woody biomass, algal biochar, and municipal solid waste (compost), respectively) [11,12,14].

Compound Name (IUPAC)	Emissions (µg/kg)	% of Total Emissions	Boiling Point (°C)	Type of VOC ^1^	Observed in Emissions from Biochar (+, −, =, Yes, No)		
					[11]	[12]	[14]
Nonanal *	2860.00	17.400	195	VOC	−	−	−
Octanal *	1480.00	9.010	171	VOC	+	−	−
Heptanal *	1180.00	7.150	153	VOC	+	−	−
butylbenzene	1030.00	6.290	183	VOC	−	−	−
Hexanal *	843.00	5.120	130	VOC	+	−	−
1-methyl-4-prop-1-en-2-ylcyclohexene	789.00	4.800	176.5	VOC	−	−	−
Benzaldehyde *	777.00	4.720	179	VOC	+	−	−
Decanal *	554.97	3.373	208	VOC	−	−	−
Toluene *	535.78	3.257	110.6	VOC	+	−	−
hexylbenzene	521.82	3.172	228	VOC	−	−	−
4,6,6-trimethyl-bicyclo[3.1.1]hept-3-ene *	408.38	2.482	155.5	VOC	−	−	−
1,3,5-trimethylbenzene	387.43	2.355	165	VOC	−	−	−
1-undecyne	373.47	2.270	195	VOC	−	−	−
2-ethyl-2,3-dihydro-1*H*-indene	342.06	2.079	-	-	−	−	−
1-ethyl-3,5-dimethyl-benzene	246.07	1.496	184	VOC	−	−	−
4,7-dimethyl-2,3-dihydro-1*H*-indene	235.60	1.432	225.9	VOC	−	−	−
1,4-xylene	225.13	1.368	138	VOC	−	−	−
1-methyl-1*H*-indene	204.19	1.241	199	VOC	−	−	−
**acetic acid** *	**197.21**	**1.199**	**118**	**VOC**	**+**	**+**	**−**
heptan-2-one *	160.56	0.976	149	VOC	+	−	−
2-methyl-5-propan-2-ylphenol	139.62	0.849	236.5	VOC	−	−	−
4-methyl-1-propan-2-ylccyclohexene *	136.13	0.827	166.8	VOC	−	−	−
Undecane *	136.13	0.827	196	VOC	−	−	−
1,3-dimethyl-2,3-dihydro-1*H*-indene	122.16	0.743	208.7	VOC	−	−	−
pyrimidine *	118.67	0.721	124	VOC	−	−	−
2-ethyl-1,4-dimethylbenzene	115.18	0.700	187	VOC	−	−	−
furan-2-carbaldehyde	113.44	0.690	162	VOC	−	−	−
1,2,3,4-tetrahydro-naphthalene	109.95	0.668	207	VOC	−	−	−
1-ethenyl-2,4-dimethylbenzene	108.20	0.658	-	-	−	−	−
1,2,3,5-tetramethyl-benzene	108.20	0.658	198	VOC	−	−	−
1,2,4,5-tetramethyl-benzene	104.71	0.636	196.5	VOC	−	−	−
1,3-xylene	99.48	0.605	139	VOC	−	−	−
pentylbenzene	97.73	0.594	205	VOC	−	−	−
2-oxopropyl acetate	95.99	0.583	175	VOC	−	−	−
Phenol *	**95.99**	**0.583**	**182**	**VOC**	**−**	**+**	**+**
1,2-diethylbenzene	92.50	0.562	183	VOC	−	−	−
2-ethyl-1,3-dimethyl-benzene	87.26	0.530	190	VOC	−	−	−
unknown isomer of ethyldimethyl benzene	85.51	0.520	-	-	−	−	−
Styrene *	75.04	0.456	145.5	VOC	−	−	−
methyl benzoate	66.32	0.403	198.5	VOC	−	−	−
6-methyl-1,2,3,4-tetrahydronaphthalene	62.83	0.382	226	VOC	−	−	−
2-ethyl-1,4-dimethylbenzene	61.08	0.371	187	VOC	−	−	−
unknown compound	59.34	0.361				−	−
2,3-dihydro-1*H*-indene	54.10	0.329	176	VOC	−	−	−
*n*-propylbenzene	52.36	0.318	159	VOC	−	−	−
1-methyl-4-propan-2-ylbenzene	50.61	0.308	177	VOC	−	−	−
1-(furan-2-yl)ethanone	48.87	0.297	168	VOC	−	−	−
2-methylpyrazine	47.12	0.286	135	VOC	−	−	−
4-methyl-2,3-dihydro-1*H*-indene	45.38	0.276	204	VOC	−	−	−
1,3-diethyl-5-methylbenzene	43.63	0.265	200.7	VOC	−	−	−
5-methyl-2,3-dihydro-1*H*-indene	41.88	0.255	204.1	VOC	−	−	−
unknown compound	41.88	0.255	-	-		−	−
dec-3-yn-1-ol	36.65	0.223	130.5	VOC	−	−	−
1,4-dimetylopirydyne	34.90	0.212	159	VOC	−	−	−
pentan-1-ol *	33.16	0.202	138	VOC	−	−	−
azulene	24.43	0.148	242	VOC	−	−	−
1-methyl-4-propan-2-yl-2-[(*E*)-prop-1-enyl]benzene	22.69	0.138	-	-	−	−	−
propanoic acid *	22.69	0.138	141.5	VOC	−	−	−
1,3-diethylbenzene	20.94	0.127	182	VOC	−	−	−
unknown isomer of diethyl methylbenzene	20.94	0.127	-	-		−	−
2,4-diethyl-1-methylbenzene	19.20	0.117	205	VOC	−	−	−
4-ethylpyridine	15.71	0.095	168	VOC	−	−	−
unknown compound	15.71	0.095				−	−
1,2,4-trimethylbenzene	13.96	0.085	168	VOC	−	−	−
1,5-dimethyl-1,2,3,4-tetrahydronaphthalene	13.96	0.085	247.5	SVOC	−	−	−
5,6-dimethyl-1,2,3,4-tetrahydronaphthalene	13.96	0.085	-	-	−	−	−
2-methylpropanoic acid	10.47	0.064	155	VOC	−	−	−
3,3-dimethyl-2*H*-inden-1-one	8.73	0.053	122	VOC	−	−	−
1-methylnaphtalene	8.73	0.053	120	VOC	−	−	−
5-methylfuran-2-carbaldehyde	6.98	0.042	188	VOC	−	−	−
2-ethylpyrazine	6.98	0.042	152.5	VOC	−	−	−
pyridine	6.98	0.042	115	VOC	−	−	−
1-methyl-2-propylbenzene	5.24	0.032	185	VOC	−	−	−
1,2-xylene	5.24	0.032	144	VOC	−	−	−
hexa-2,4-diene, (*E,E*)-	5.24	0.032	82	VVOC	−	−	−
1-phenylethanone	1.75	0.011	202	VOC	−	−	−
2,5-dimethylpyrazine	1.75	0.011	155	VOC	−	−	−
4-ethyl-1,2-dimethylbenzene	1.75	0.011	236.5	VOC	−	−	−
cumene	1.75	0.011	153	VOC	+	−	−
pentanoic acid *	1.75	0.011	110.5	VOC	−	−	−
1,4-diethyl-2-methylbenzene	0.10	0.001	207	VOC	−	−	−
2-methoxyphenol *	0.10	0.001	205	VOC	−	−	−
3-methylbutanoic acid	0.10	0.001	176	VOC	−	−	−
5-methyl-1,2,3,4-tetrahydronaphthalene	0.10	0.001	234	VOC	−	−	−
Total	16,452.46	-	-	-	−	−	−

^1^ —according to [16], where VVOC—very volatile organic compounds (0–100 °C), VOC—volatile organic compounds (100–240 °C), SVOC—semi-volatile organic compounds (240–400 °C); bold font = common compounds found in at least two other studies; * Identified using analytical standards.

## Supplementary Materials

The following files have been submitted as supplementary materials in zipped folder “supplementary materials.zip”: explanatory file “readme.docx”, raw data in files “CRDF MS raw data.jdx; CRDF MS raw data.csv; CRDF peaks raw data.xlsx” and tables (Tables S1 and S2) in the file “Tables.xlsx”.
