# Supplementary material for: Quantification of VOC Emissions from Carbonized Refuse-Derived Fuel Using Solid-Phase Microextraction and Gas Chromatography-Mass Spectrometry
Source: Molecules. 2018 Dec 5;23(12):3208. doi: 10.3390/molecules23123208 (PMC6321041; doi:10.3390/molecules23123208)
Supplement: Supplementary file 1 [file molecules-23-03208-s001.zip › Supplementary materials/Readme.pdf]

"Supplementary materials" explanation:

All data were obtained after analyses on Varian Saturn2000 and processed with using Varian MS Workstation in .SMS extension.

Therefore, all data were converted with using ACD/Spectrus Processor 2017.2.1 into .JCAMP (.jdx) extension which may be open by software's like Mnova MS 12.0.1. This file is named "CRDF MS raw data.jdx". This file has been converted to csv extension named "CRDF MS raw data.csv".

Moreover, all raw data from analyses are collected into Excel file "CRDF peaks raw data.xlsx".

Tables 1 and 2 from the manuscript were included in the Excel file "Tables.xlsx".

Concluding, following files have been submitted as supplementary materials in zipped folder "supplementary materials.zip":

Readme.docx

- CRDF MS raw data.jdx
- CRDF MS raw data.csv
- CRDF peaks raw data.xlsx
- Tables.xlsx
